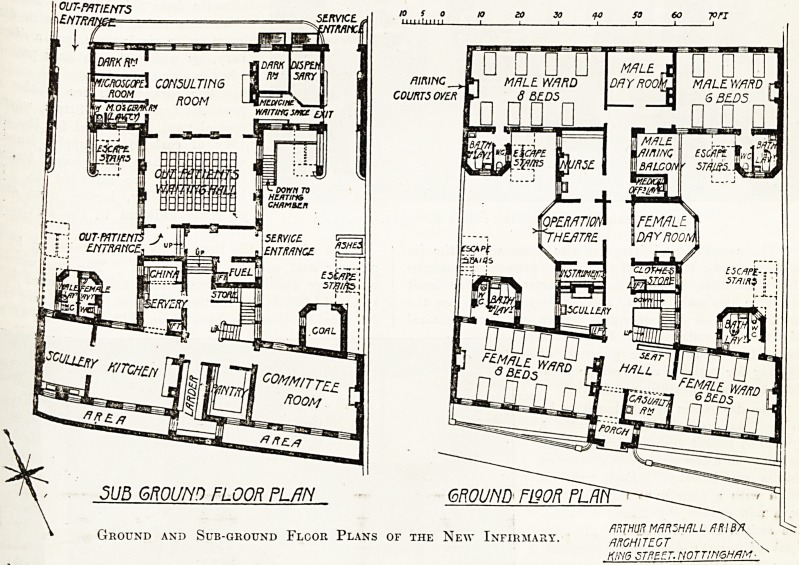# The Nottingham and Midland Eye Infirmary

**Published:** 1912-07-27

**Authors:** 


### HOSPITAL ARCHITECTURE AND CONSTRUCTION.

[Communications on this subject should be marked "Architecture" in the left-hand top corner of the envelope.]

### The Nottingham and Midland Eye Infirmary.

It would appear from the description which has been furnished to us that the site upon which this hospital stands has a somewhat steep fall from north to south. Hence it comes about that the entrances at the north end are on the sub-basement level and the main entrance at the south end on the ground-floor level.

The north front abuts upon Oxford Street and the south front upon Rope Walk. The out-patient entrance is on the Oxford Street side at the sub-ground level; from a covered porch the out-patients pass into a waiting-hall capable of seating about 100 patients, and from thence into one large consulting-room, off which are two dark-rooms, a microscope-room, and a cloak-room and lavatory for the medical officer. On the east side of the consulting-room is a small lobby for patients waiting for medicine, and the dispensary, and immediately out of this lobby is the out-patient exit into Oxford Street at the south-east corner.

Ground and Sub-ground Floor Plans of the New Infirmary.

Arthur Marshall A.R.I.B.A. Architect King Street. Nottingham.

The sanitary offices are arranged for male and female patients in the entrance yard. The remainder of the sub-ground floor is occupied by the kitchen offices and a committee room, and in a basement below this storey is the heating chamber.

The ground-floor plan contains two wards for women of eight and six beds respectively, and two similar wards for men, and for each sex is provided a day room, and in addition for the men is an open balcony. The female day room is also so arranged that by a system of sliding windows the whole room can be in a few seconds converted into a sun balcony.

The sanitary offices are built out in annexes, the lobbies to which give access to the escape staircases, of which there are four sets. A portion of the entrance-hall is separated off to form a casualty room, where minor accidents to the eye can be immediately attended to.

In the centre of the building is an operation room, with a small room for instruments adjoining. There is also a nurses' room, a ward scullery, and a patients' clothes store on this floor. The floor above contains accommodation for the staff, including serving room, nurses' dining and sitting rooms, matron's quarters, nurses' and servants' bedrooms, with bath-rooms, lavatories, etc. This part of the building is so constructed that in the event of additional accommodation being required an additional storey can be built without interfering with the work of the institution.

The building has evidently been very carefully thought out, and should prove a very valuable addition to the hospital work of Nottingham. The architect, by whom the building has been designed, is Mr. Arthur Marshall, of Nottingham.

## Figures and Tables

**Figure f1:**